# Autonomous mining through cooperative driving and operations enabled by parallel intelligence

**DOI:** 10.1038/s44172-024-00220-5

**Published:** 2024-05-31

**Authors:** Long Chen, Yuting Xie, Yuhang He, Yunfeng Ai, Bin Tian, Lingxi Li, Shirong Ge, Fei-Yue Wang

**Affiliations:** 1 Institute of Automation, Chinese Academy of Sciences, Beijing, China; 2 Waytous Inc., Beijing, China; 3 The School of Mechanical Electronic and Information Engineering, China University of Mining and Technology Beijing, Beijing, China; 4 Institute of Artificial Intelligence and Robotics, Xi’an Jiaotong University, Xi’an, China; 5 The School of Computer Science and Engineering, Sun Yat-sen University, Guangzhou, China; 6 Department of Computer Science, University of Oxford, Oxford, UK; 7 The School of Artificial Intelligence, University of Chinese Academy of Sciences, Beijing, China; 8 The Elmore Family School of Electrical and Computer Engineering, Purdue University, Indianapolis, USA

**Keywords:** Electrical and electronic engineering, Mechanical engineering

## Abstract

Autonomous mining is promising to address several current issues in the mining sector, such as low productivity, safety concerns, and labor shortages. Although partial automation has been achieved in some mining operations, fully autonomous mining remains challenging due to its complexity and scalability in field environments. Here we propose an autonomous mining framework based on the parallel intelligence methodology, employing self-evolving digital twins to model and guide mining processes in the real world. Our framework features a virtual mining subsystem that learns from simulating real-world scenarios and generates new ones, allowing for low-cost training and testing of the integrated autonomous mining system. Through initial validation and extensive testing, particularly in open-pit mining scenarios, our framework has demonstrated stable and efficient autonomous operations. We’ve since deployed it across more than 30 mines, resulting in the extraction of over 30 million tons of minerals. This implementation effectively eliminates the exposure of human operators to hazardous conditions while ensuring 24-hour uninterrupted operation.

## Abstract (web summary)

Long Chen and colleagues show a fully autonomous open-pit mine. Heterogenous machinery and tasks are coordinated using parallel learning and digital twins.

## Introduction

The mining industry is a critical sector that provides essential raw materials for engineering advancements, serving as one of the most essential energy sources for the whole world, and boosting the global economy accordingly. Nevertheless, the traditional mining industry has been always facing various challenges, including adverse working conditions, labor shortages, and re-occurring mining accidents. In the meantime, due to the ever-increasing global energy demand, there is a critical need to enhance the productivity of mineral resources. Automating the traditional mining industry with artificial intelligence or other digitalization techniques thus becomes a prerequisite and necessary. It is expected to improve mining productivity and efficiency, enhance workforce safety, and minimize the environmental impact brought by mining operations^1^. Such automated mining systems are called “Autonomous Mining”.

In recent years, some prior works have been proposed to partially automate the mining processes from different perspectives, ranging from autonomous truck development^2–4^, remotely controlled excavation system design^5^, automated drilling and blasting^6^ to mining rescue robots^7^ and 3D geographic modeling for mining areas^8^. Although these preliminary attempts showed success in their own local tasks under simplified and idealized scenarios, it still remains extremely challenging to automate the mining processes that can easily scale up to large-scale, heterogeneous, and complex scenarios where all mining facilities need to work collaboratively and synergically in a timely manner. Several challenges must be overcome before fully autonomous mining can be achieved.

**Fig. 1:**
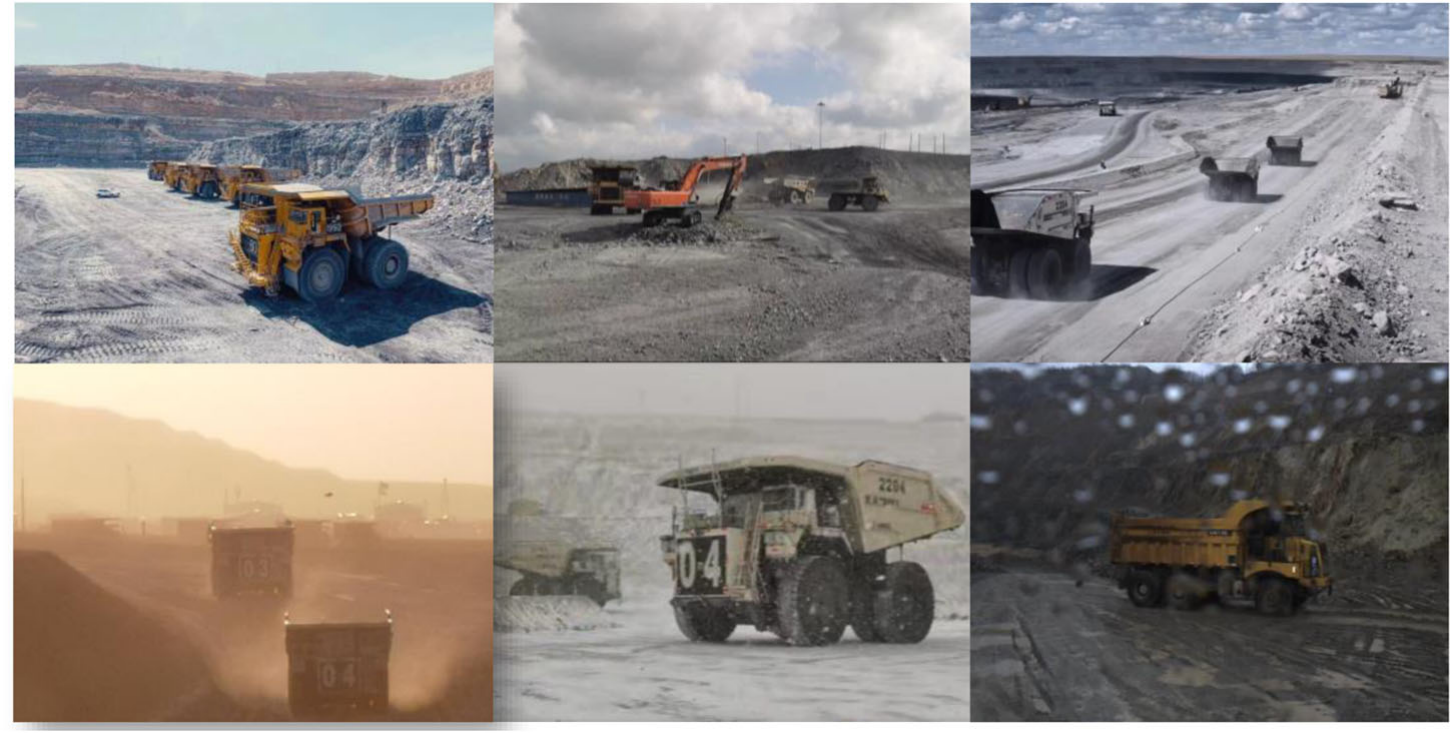
Mine environments. The mine environments present a range of challenges, including uneven terrain, floating dust, heavy machinery, varying illumination, and changing weather conditions.

Challenge 1: Generalization issues in field environments. Mine terrains are often uneven and irregular, the floating dust and fumes seriously reduce machines’ visibility. Moreover, the adverse and highly diverse weather conditions have enormously increased the mining difficulty^9,10^ (as shown in Fig. 1), requiring the whole mining system to be able to work reliably and robustly across all weather conditions.

Challenge 2: Complex collaboration among devices. Autonomous mining is a complex system in which a group of automated machines (or mining robots) are participating in different tasks simultaneously, including excavation, transportation, ventilation, and maintenance, etc. All the involved machines need to timely negotiate and communicate with each other in a real-time manner so as to collaboratively finish one task, which precisely illustrates the characteristics of large-scale, heterogeneous, and complex task-oriented multi-robot systems. Multi-robot systems have been studied extensively^11,12^, with research topics spanning from self-organizing bionic clusters^13–16^ to hierarchical multi-robot systems^17–19^. However, constructing such a complex robot system remains an unresolved problem^12,20^.

Challenge 3: Adequate pre-validation. Inappropriate mining operations can potentially cause huge economic losses and even fatalities. Therefore, each operation to be executed by each machine needs to be adequately validated and tested to ensure its safety and feasibility. Moreover, such validation should be completed through a platform that is cost-effective and reversible (i.e., we can test as many cases as we need). However, in mines, setting up extensive physical field tests is impractical with high cost, high-security risk, and low efficiency. Moreover, the most challenging and risky situations are less frequently encountered and thus impossible to adequately test in the physical world. Simulation can provide massive test cases^21–23^, but it remains unsolved to close the sim-to-real gap, especially for a large-scale integrated system^24,25^.

**Fig. 2:**
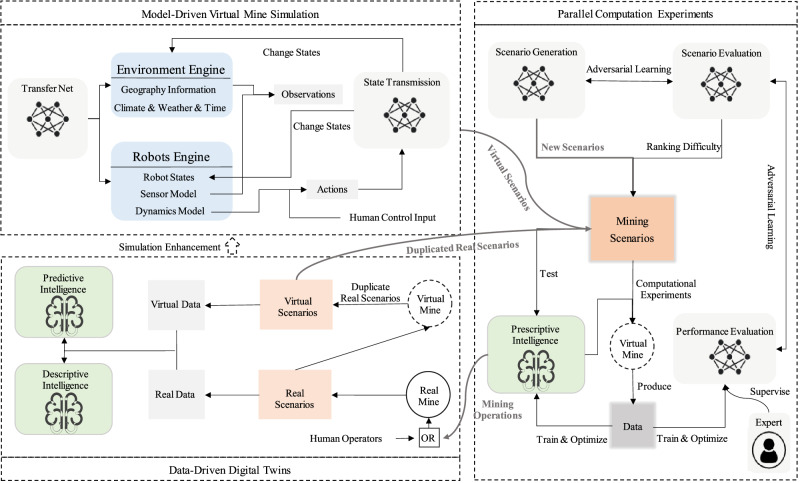
Overview of the parallel mining conceptual framework. Integrating model-driven virtual mine simulation, data-driven digital twins, and parallel computation experiments for generating scenarios in mining algorithm training and testing.

To address the aforementioned challenges, we propose an autonomous mining framework based on the parallel intelligence methodology^26^ that: (1) enables large-scale multi-agent mining with little human intervention, (2) is capable of handling complex mining cases and diverse weather conditions, and (3) is self-evolutionary so that it performs better and better as more mining work has been completed. Building upon our previous work^27^ that introduced a basic end-to-end automated mining paradigm highlighting the collaborative control enabled by the Internet of Things in mining devices, this paper further extends the framework and demonstrates a notable step forward in the practical realization of parallel intelligence. Specifically, the framework that achieves autonomous mining via parallel intelligence, named “Parallel Mining”, consists of three main components (as shown in Fig. 2).

The first component, model-driven virtual mine simulation, involves manually building an environmental model engine for mining, as well as sensors and dynamics models for mining machinery (robots). This component also includes defining the state-transition paradigm for the virtual mine.

The second component, data-driven digital twins, collects data from sensors embedded in on-site equipment (such as mining robots and environment monitor devices) and control commands to replicate real-world scenarios in the virtual mine. By using real-world data as supervision, this component enhances the descriptive and predictive intelligence of the virtual mine, particularly improving the simulation models and state-transition paradigms.

The third component, parallel computing experiments, uses a scenario library to evaluate and optimize mining operations in the virtual mine. This library includes real-world scenarios, hybrid scenarios that combine human input and virtual engine responses, and generated virtual scenarios. This module generates prescriptive intelligence from a large number of computational experiments that can be applied to real mining operations.

We have successfully implemented Parallel Mining into the “stripping-transportation-dumping” process of open-pit mines. The developed system oriented by this framework has been put into long-term usage in Yimin Open-pit Mine, located in Inner Mongolia, China, to comprehensively monitor and evaluate its performance. The system can achieve uninterrupted runs for 24 h over a long period, demonstrating comparable efficiency to human operators. This case study initially proves the feasibility and effectiveness of Parallel Mining. The results of this research are expected to address the bottleneck of autonomous mining and provide a new basis for the management and control of complex systems.

## Results

Oriented by the Parallel Mining framework, we developed an autonomous mining system for open-pit mines, named YuGong (drawing inspiration from an old Chinese legend, a foolish man in his 90-year age is determined to remove obstructed mountains with strong willingness and perseverance). We comprehensively test YuGong’s stability, robustness, and efficiency in Yimin Open-pit Mine, an area of Inner Mongolia Autonomous Area, China (covering a vast area of 42.35 km^2^ and situated at high altitudes with an extremely cold climate, where frequent rainy, snowy, or foggy days occur). We demonstrate that by parallel computation experiments, mining operations can be tested and evaluated efficiently and safely in a virtual mine meta-universe, and then be successfully applied to real-world mines (refer to Supplementary Movie 1 for a comprehensive real-world demonstration).

### System design

**Fig. 3:**
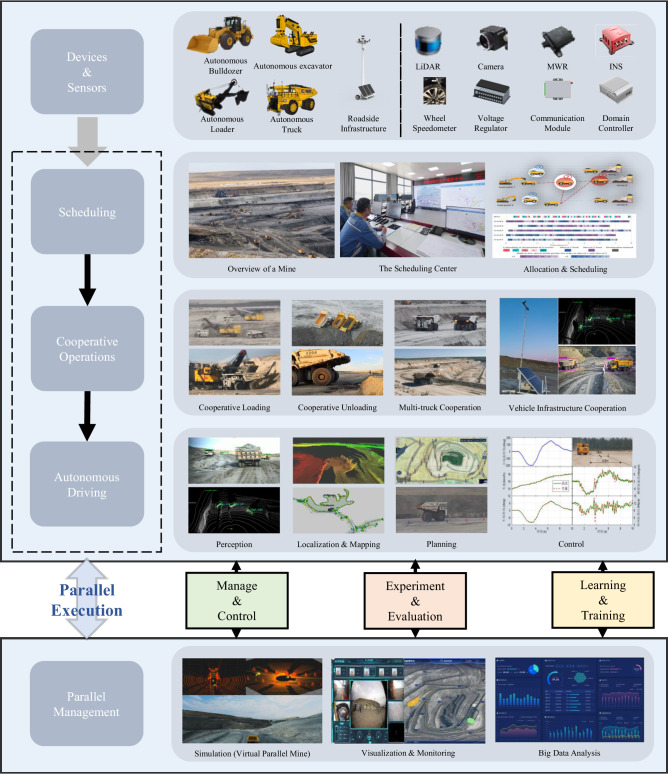
The autonomous mining system for open-pit mines. LiDAR Light Detection and Ranging, MWR Millimeter Wave Radar, INS Inertial Navigation System.

The system design of YuGong is depicted in Fig. 3. On the physical mining site, we configure multiple mining vehicles (in our case, mining trucks, excavators, bulldozers, and loaders). To achieve autonomous mining, we equip each individual vehicle with various sensors that have been widely used and verified in autonomous driving^28,29^, including LiDARs, cameras, millimeter Radars, wheel speedometers, and GPS/INS combined positioning (refer to Supplementary Fig. 2 for specific hardware configuration on mining devices). Our on-vehicle sensors collect egocentric data that inform decisions about the vehicle’s actions. This data is also used to reflect the real-time representation of the mining site, which is further synchronized with our virtual platform. In addition, we have installed roadside infrastructures with cameras and LiDARs to supplement perception, reducing blind spots and improving safety. Both the vehicle and roadside units are equipped with communication devices for vehicle-to-everything communication^30^, facilitating information sharing and collaboration.

The system comprises three main modules: the autonomous driving module, the scheduling and cooperative operations module, and the parallel management module. The autonomous driving module based on the robot operating system architecture^31^, enables mining vehicles to perform tasks autonomously and is fundamental to the autonomous mining system. The cooperative operations module takes charge of tasks dispatching and guides vehicles to conduct coordinated operations. The parallel management module is responsible for maintaining a data center and a virtual mine engine. This involves collecting, processing, and analyzing operational data, as well as providing testing, optimization, and early warning of algorithms in the previous modules. In addition, the parallel management module monitors the operational status of the hardware. During mining, mining vehicles follow commands from the cooperative operations module and the autonomous driving module, with the cooperative operations module taking precedence. Algorithms of these modules are deployed after passing tests in the virtual mine.

The integrated hierarchical system design aims to enhance system-level stability. The scheduling and cooperative operations module handles part of the decision-making, reducing vehicle-side computational demands. This allows essential task execution, even if the autonomous driving module fails in a non-adversarial environment. In addition, increasing the level of collaboration can compensate for the insufficient robustness of self-driving algorithms. For instance, roadside infrastructures can inform obstacles ahead when the truck’s field of view is obstructed or the online perception unit fails due to environmental noise. The autonomous driving module is also able to flexibly execute tasks if higher-level modules malfunction. The parallel management module monitors device status and predicts the effectiveness of instructions to prevent accidents and initiate manual intervention in the event of the scheduling and cooperative operations module and autonomous driving module failures. Furthermore, the parallel management module collects online data to improve simulation, support advanced algorithm testing, and reduce algorithmic issues.

### Virtual mine and parallel testing

The virtual simulation engine is a critical component of our Parallel Mining framework. We have created a virtual mine engine for open-pit mining operations in YuGong, which is incorporated in the parallel management module. This section showcases the engine’s performance, including physical simulation results, reality replication capabilities enabled by digital twins^32^, and scenario library construction capabilities for testing.

**Fig. 4:**
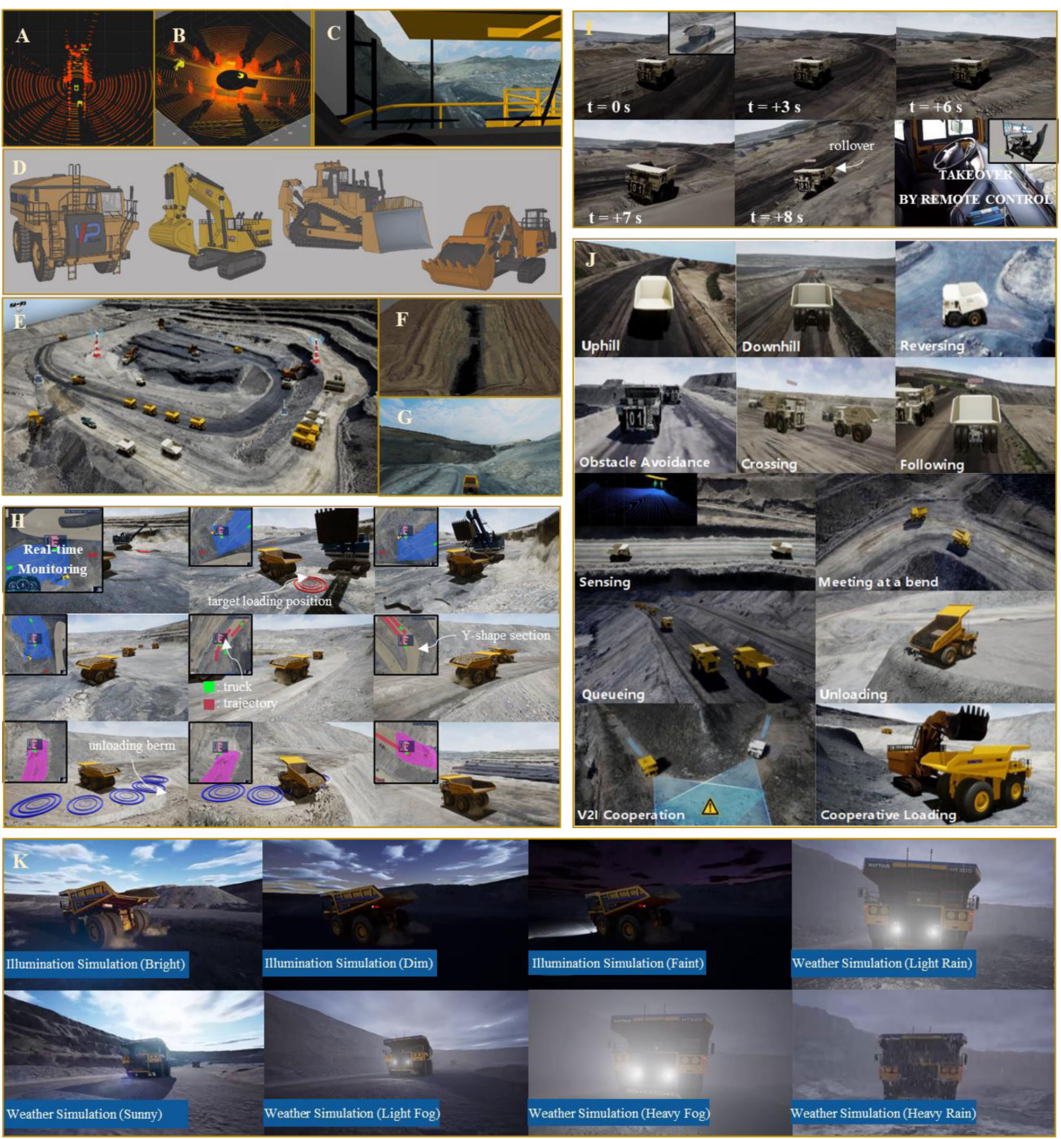
Testing scenarios generation through virtual-real interactions. **A**–**G** Virtual entities in the mine meta-universe. Three essential types of models are involved in the mine meta-universe, including sensor models (**A**–**C**, virtual sensor observations), mining vehicle models (**D**), and the mine model (**E**–**G**, the overview and details of the mine model). **H** One complete transportation process with interactions between the simulation and the real world. **I** Ultra-real-time simulation. **J** Basic functional tests in the virtual mine. **K** Illumination simulation and weather simulation.

Figure 4A–G shows a set of virtual entities in the virtual mine. The virtual mine for open-pit mining management consists of three types of entity models: the mine environment, mining vehicles, and sensors. We achieve the validity and fidelity of the simulation by 1:1 modeling of the physical counterparts. The mine model restores the environment and achieves pixel-level simulation by rendering. Mining vehicle models replicate the dynamics model, body dynamics model, hydraulic machine steering system, and tire dynamics model to give a realistic physical control effect. The placement of virtual sensors is identical to that of physical on-site sensors, and the measurements reflect the physical properties of real sensors.

Based on entity models, the mining activities in the physical mine can be reproduced in the virtual mine through the constructed data channels from reality to virtualization. In this case, the virtual vehicles follow the same control instructions as their physical counterparts. With accurate descriptive models, the virtual mining trucks should keep consistent motion patterns and identical observations with the real ones. Figure 4H shows the replication of a complete transport process, including multiple complex behaviors of the mining truck, which demonstrate the mine meta-universe’s ability to duplicate reality. The truck departs from the preparation area and enters the loading yard traditionally. Before reaching the idle forklift, the truck reverses to the target dumping position of the forklift mechanical arm and waits for the loading to be completed. After loading is completed, the truck leaves the site and drives along the planned route to the unloading destination. During this period, it drives through the uphill and downhill sections, curve sections, and Y-shaped sections with heavy loads, and avoids conflicts with other trucks. When the unloading site is reached, the truck backs up approaches the designated unloading berm, and automatically lifts the hopper to incline materials. After unloading, the truck leaves the unloading site and continues driving to the next loading point assigned. The animated demonstration can be found in Supplementary Movie 2.

Along with replicating real-world scenarios, the virtual engine can predict future outcomes. Figure 4I demonstrates an ultra-real-time warning scenario, where the virtual truck follows the control of its physical counterpart and is predicted to collide with the mountain causing a rollover in the near future. An emergency stop will be enforced on the real truck immediately by remote control.

The virtual engine in Parallel Mining is designed to learn and generate new scenarios by replicating and predicting. As a result, it not only reproduces real-world scenarios but can also generate virtual testing scenarios. Figure 4J shows multiple types of test scenarios used to test the basic functions of the autonomous mining algorithms. Figure 4K presents generative simulation results in different weather and illumination conditions.

### Field testing

To evaluate the real-world performance of the algorithms after passing the parallel testing and demonstrate the robustness of the system, we set up several special testing scenarios in a mining test field. There are seven groups of testing scenarios, with three dedicated to assessing autonomous driving performance, three dedicated to evaluating cooperative operation performance, and one focusing on adverse conditions.

**Fig. 5:**
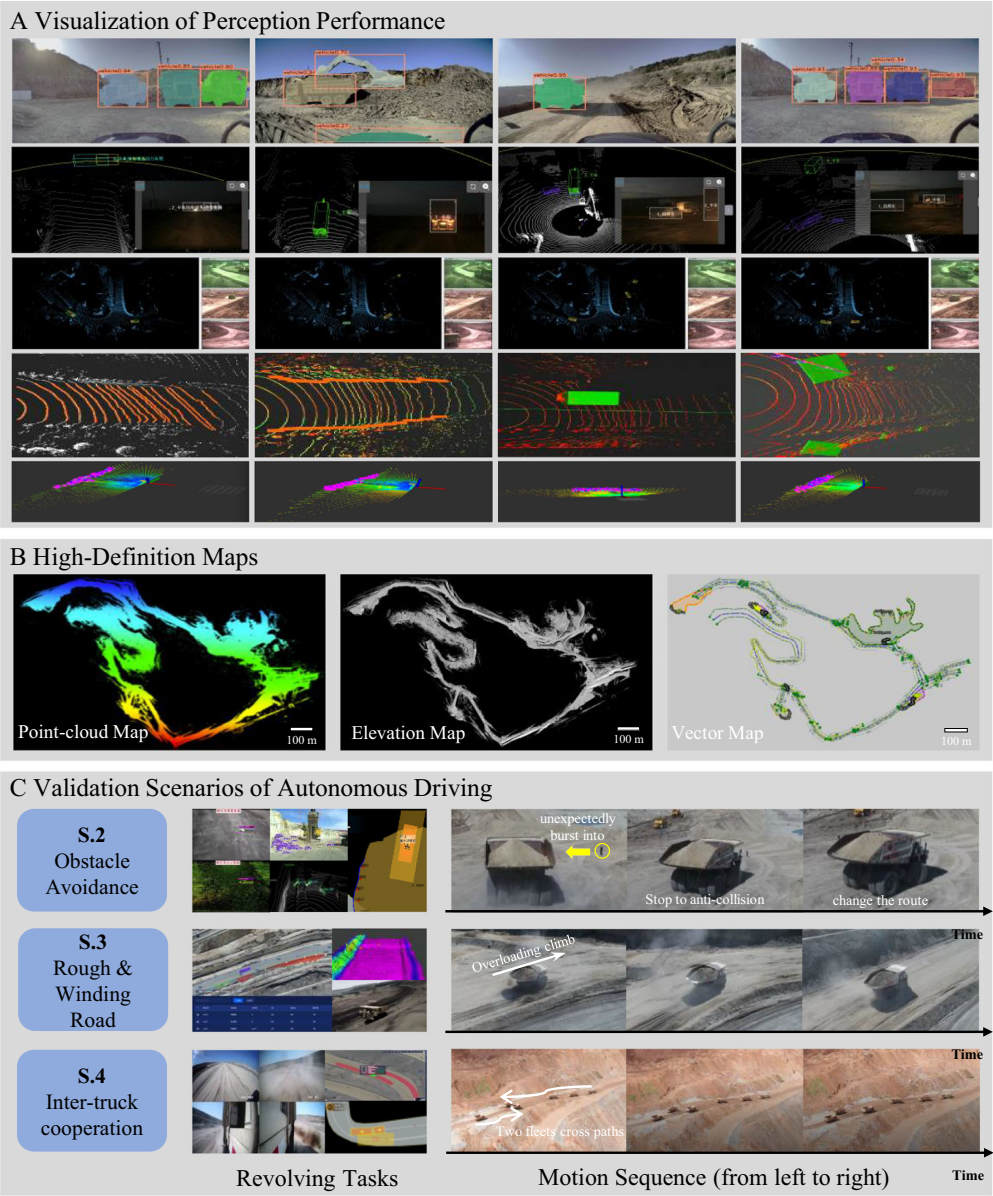
System validation in autonomous driving scenarios. **A** Visualization of perception performance. Rows from the top to the bottom are the results of vehicle detection during the day, vehicle detection at night, vehicle detection with LiDAR on roadside units, road detection, and berm detection in dump areas. **B** High-definition maps of an open-pit mine consist of a point-cloud map, an elevation map, and a vector map. **C** Real-world scenarios for validating autonomous driving capabilities.

Scenario 1: Fundamental self-driving. In YuGong, each individual mining vehicle conducts the mining operations both independently and collaboratively. The prerequisite for each individual mining vehicle is to work autonomously. To this end, we set up all the necessary self-driving relevant technologies. During the mining transportation process, the mining site’s 3D high-precision map is scanned and updated with a virtual metaverse. During the mining process, the 3D high-precision map is used by autonomous driving, Path Planning module to plan an efficient route for each working mining truck, and the Control module accordingly sends commands to maneuver each mining truck. Figure 5A, B demonstrates the perception results and high-definition maps, respectively. Table 1 illustrates the quantitative comparative results of our system’s segmentation performance in mining scenarios, showcasing superiority over other state-of-the-art methods, primarily attributed to the data-level enhancement of model generalization facilitated by our parallel system’s scenario data generation pipeline.

**Table 1:** Segmentation results of *YuGong*, compared to state-of-the-art methods on the AutoMine dataset^2^ (a mine dataset)

Category	Sky	Massif	Tussock	Road	Road edge	Mine truck
SegFormer^51^	99.8	84.83	62.98	88.86	68.96	90.05
Mask2Former^52^	99.81	86.46	63.58	88.65	70.84	89.22
Swin^53^	99.74	82.41	54.5	86.37	63.82	80.85
FCN^54^	99.69	79.78	29.48	86.23	62.03	74.16
Segnext^55^	99.55	81.22	56.1	86.55	65.4	69.2
Segmenter^56^	98.81	74.63	49.6	78.23	56	76.09
UPerNet^57^	99.72	80.51	12.04	86.03	64.86	81.37
deeplabv3+^58^	98.9	79.19	50.96	83.88	63.46	73.81
U-Net^59^	99.43	74.42	35.22	83.99	54.31	68.75
*YuGong*	**99.86**	**92.67**	**64.24**	**95.59**	**81.46**	**93.68**

The evaluation covers primary driving-related areas and elements in mines, with specific emphasis on “Road Edge” and “Mine Truck” as they serve as crucial indicators for autonomous driving in mining operations.

^*^Numbers highlighted in bold represent the best results.

Scenario 2: Obstacle avoidance. Safety is paramount during mining operations, and the system must be able to handle emergencies, particularly when obstacles suddenly appear in the working path. To evaluate the system’s obstacle avoidance capabilities, we designed a series of tests using various obstacles such as rocks, traffic cones, dummies, and people crossing the truck’s path. Figure 5C demonstrates an example of the truck avoiding a worker crossing its path. When the system detects an unknown person entering the truck’s path, the truck immediately stops to avoid a collision. If the person stops in front of the truck, the system replans its route. The success of these tests required collaboration among multiple units and comprehensively evaluated the stability of perception, planning, and safety control. Specifically, the irregular, multi-scale objects in the mining environment present significant challenges for perception. To ensure complete obstacle detection, the system merges results from on-vehicle sensors and roadside infrastructure.

Scenario 3: Rough and winding roads. Unlike structured urban roads, the roads in mining areas are irregular. Therefore, mining vehicles must have the ability to travel smoothly on muddy, rugged, and uneven roads. This set of tests primarily evaluated the system’s control performance. Figure 5C illustrates a sample scenario where a truck carrying a heavy load travels up a rough and winding slope. To achieve smooth control, real-time perception, mapping, and planning are all required. Perception provides real-time flatness detection results, maps offer information on the slope and speed limit of the road section, and planning outputs trajectories and speeds based on the above information. Additionally, the planned commands’ potential future situations are predicted in advance through an online reference on the simulation platform. If there is a risk of overturning, the truck will be stopped or remotely taken over in time.

Scenario 4: Inter-truck cooperation. In mining areas, traffic markings and signs commonly seen on urban roads are absent, requiring mining trucks to collaborate flexibly with each other. To evaluate the coordination ability among trucks, tests on inter-truck cooperation were conducted on different road segments, including platooning, crossing, and merging. Figure 5C illustrates a scenario where two truck fleets from opposite directions meet. These tests also involve multiple modules. The scheduling and cooperative operations module designs collision-free paths between fleets in advance. The planning unit controls the following distance of trucks and ensures collision-free meetings. The control unit further enhances platooning safety by limiting the safety distance. To avoid road damage caused by repeated rolling of single paths, the anti-rutting unit ensures evenly distributed rolling paths on the road.

**Fig. 6:**
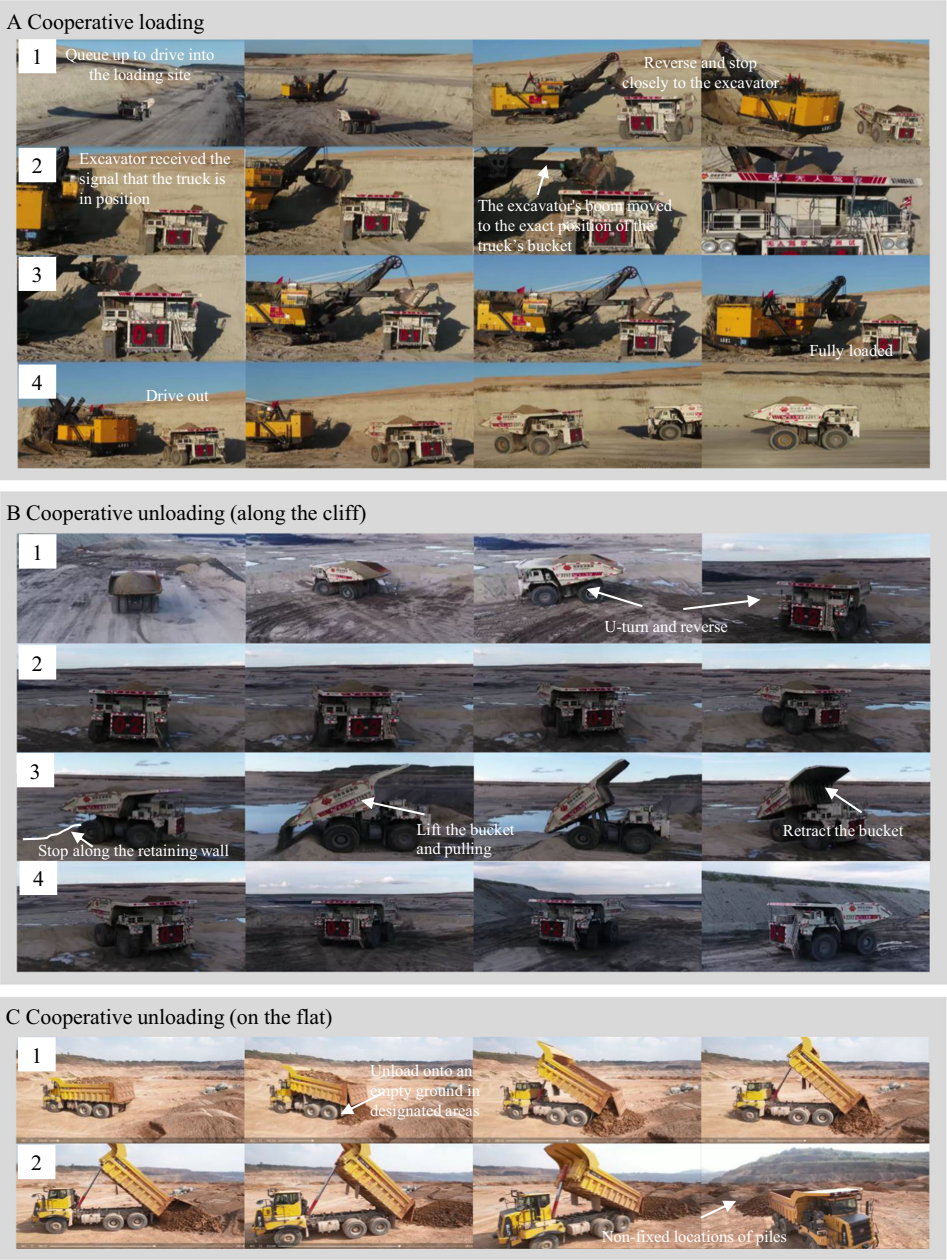
Cooperative operations in real-world scenarios. **A** 1–2: The autonomous truck drives into the entry point of the loading area and reverses to the loading point. 3–4: The autonomous truck loads materials and drives out of the loading area. **B** Unloading materials along the cliff. 1–2: The autonomous truck drives into the entrance point of the dump and reverses to the unloading point. 3–4: The autonomous truck unloads materials and drives out of the dump. **C** Unloading materials on the flat.

Scenario 5: Cooperative loading. The trucks should queue up to enter the loading site in order and cooperate with the excavator to load materials autonomously. This scenario is set to test YuGong’s performance at loading sites, the area with the most frequent collaboration among devices. The multi-truck cooperation ensures the ordered entrance and exits to improve safety. The localization unit on the excavator boom tracks the real-time position of the bucket. Autonomous driving on the truck ensures its accurate stopping at the loading point indicated by the position of the bucket. When the truck is fully loaded and exits the site, the vehicle-to-everything communication platform informs the excavator to wait and instructs the next truck in the queue to be in place. Figure 6A demonstrates the process of cooperative loading.

Scenario 6: Cooperative unloading. The mining truck needs to unload materials along one cliff or cooperate with the bulldozer to unload materials on the ground. Similar to collaborative loading, this scenario involves technologies including multi-truck cooperation, fixed-point parking, and vehicle-to-everything communication. However, unloading sites pose even greater risks due to the continuous movement of the cliff edge caused by the accumulation of tilted materials, and the typically soft ground in the unloading sites. Potential dangers include rollovers over the retaining wall, vehicle sinking, and collisions resulting from dynamic changes. Therefore, this scenario is a severe test for perception, planning, and control, as well as map service and resource dispatching. Figure 6B, C demonstrates the process of cooperative unloading.

Scenario 7: Adverse operational conditions. We provide field operation videos under this set of scenarios (see Supplementary Movie 3), including snowy conditions, high dust environments, slippery snow-covered roads, and low-light nighttime operations. Due to limited data availability and the inherent risks associated with on-site work in these complex conditions, training for such scenarios primarily takes place within parallel simulation systems (these virtual scenarios are set up as shown in Fig. 4). It can be observed that the system demonstrates stable operation in these complex conditions. It is worth noting that a more conservative strategy is employed in such situations, leading to a reduction in the speed of the mining truck, aimed at minimizing the occurrence of accidents.

### Long-term performance

Apart from the aforementioned scenario-specific tests, we further tested YuGong’s efficiency and stability over a long period of time on Yimin open-pit coal mine site. We conducted multiple rounds of testing on a fleet of five 220-ton load capacity mining trucks, one 35 cubic meters electric shovel, and one bulldozer. These tests were carried out in a long-term collaborative effort under the management of the YuGong platform, with the trucks being fully loaded and operated autonomously.

We found that YuGong demonstrates higher mining efficiency compared to human operators. As shown in Table 2, the average loading and unloading times are about 5 min and 3 min, respectively, achieving about 10% higher efficiency than manual operations due to the cooperative system that eliminates cumbersome manual position alignment. Although there is still room for improvement, the operation time is intentionally limited due to the high-risk nature of the loading and unloading sites. Autonomous mining also demonstrates clear overall efficiency advantages. With flexible planning and control resulting in faster travel speeds than manual operation, the speed limit for safety can be increased by 33% for empty trucks and 10% for fully loaded trucks. Moreover, precise scheduling with velocity planning reduces excavator idle time and increases transportation rounds per day. In addition, autonomous vehicles can operate continuously, unlike human-operated teams that require 2 h of shift change time. These factors contribute to a 35% increase in the maximum number of daily transportation shifts per truck for autonomous driving compared to human operations.

**Table 2:** Evaluation of system performance in a long period

Average operation time per shift (Human v.s. *YuGong*)	Loading materials	300 s	265 s	11%↓
	Unloading materials	180 s	162 s	10%↓
Efficiency analysis per day (Human v.s. *YuGong*)	Maximum speed (unloaded)	30 km/h	40 km/h	33%↑
	Maximum speed (fully loaded)	30 km/h	33 km/h	10%↑
	Number of shifts	26	35	35%↑

Throughout the testing period, we successfully navigated extreme and challenging environmental conditions such as severe cold weather (down to −42 °C), heavy snow, fog, rain, high levels of dust, and strong vibrations in the Yimin mining area. During the long-term autonomous mining testing, YuGong is capable of achieving 24-hour uninterrupted autonomous mining without any human intervention. Further, long-term statistic shows that using the autonomous mining system can save over 12% of fuel consumption per ton of minerals produced compared to manual operations. The primary source of fuel savings stems from our scheduling approach that incorporates velocity, which has been proven advantageous in reducing fuel consumption in a simulation setting in our previous work^33,34^. In addition, autonomous driving tends to prioritize selecting the shortest route due to the availability of high-precision maps, resulting in enhanced efficiency compared to human driving. Moreover, as indicated in Table 2, collaborative autonomous operations facilitate expedited coordination during the loading and unloading process compared to manual docking procedures, thereby reducing fuel consumption associated with idling and waiting.

## Discussion

Our results demonstrated a promising advancement in Autonomous Mining. In all demonstrated scenarios, the proposed system successfully overcame challenges faced by robots in the field and enhanced efficiency through cooperative operations. In addition to the Yimin mine, where the comprehensive test of YuGong was conducted, the system has been successfully deployed in more than 30 mines and completed over 4.1 million kilometers without any major accidents (for more information on YuGong’s application in various mining contexts, please refer to Supplementary Discussion, including Supplementary Table 1 and Supplementary Fig. 1). Overall, it effectively eliminates human operators from hazardous mining conditions and achieves uninterrupted operation for 24 h.

While the system currently demonstrates satisfactory performance, it offers only initial validation of the Parallel Mining framework’s feasibility and effectiveness. It’s essential to highlight that open-pit mining, though a valuable demonstration, represents a simpler scenario in the mining industry compared to the more complex underground mining. In addition, the adaptation process during system migration to various mining sites, requiring fine-tuning with regional data, currently presents a cumbersome start-up procedure. How to finally attain universal autonomous mining intelligence that is plug-and-play deserves further investigation. The recent emergence of large foundation models has garnered significant public attention, and developing a unified foundation model specifically tailored for mining purposes could offer a promising solution in this domain.

The success of our system may extend beyond mining, serving as a catalyst for the emergence of self-evolving digital twin systems across diverse industrial sectors and enhancing overall control capabilities. This shift towards intelligent automation holds immense potential for widespread improvements in safety, energy efficiency, and environmental impact reduction. Moreover, integrating societal information encompassing social psychology, industry chains, market trends, and policies into our parallel framework has the potential to break down existing isolation among industry systems, paving the way for establishing an interconnected social-industrial system that promotes global sustainability through a more rational and sustainable resource allocation on a macro scale.

## Methods

### System engineering

Autonomous mining presents a sophisticated engineering system, as depicted in Fig. 3, comprising various functionalities, including perception, localization, mapping, planning, collaborative perception, and collaborative scheduling. The algorithms utilized in the YuGong system are built upon our prior research efforts^2,33–44^. These specialized algorithms tailored for mining environments have exhibited superior performance when compared to other state-of-the-art methods in specific subtasks. For instance, our dynamic mapping method effectively addresses the challenge posed by frequently changing operational areas within mines^43^, while our scheduling approach integrates map data and velocity planning to navigate the rugged terrain of mining environments^33,34,39^. To bolster safety in demanding segments, we incorporate roadside units via sensor fusion to enhance hazard perception capabilities^41^. More detailed explanations of these algorithms are available in the respective publications.

In this study, we integrate the sub-modules into a comprehensive system through parallel intelligence and scenario engineering. By implementing a data generation loop that progresses from a small amount of real-world data to large-scale data within the parallel system, we further scrutinize and optimize the overall performance, ensuring complete stability and reliability. While our system currently employs specific sub-module algorithms, they are interchangeable within the core parallel mining framework.

### Mining via parallel intelligence

Based on the pre-constructed physical world mining infrastructure and the model-driven virtual mining metaverse, we use parallel learning^25^ to achieve autonomous mining. Specifically, it comprises three main components.

#### Descriptive learning

In order to instantiate the virtual metaverse to resemble the physical world mining situation, we adopt descriptive learning. Given a proper initialization strategy, the virtual metaverse builds up every essential virtual mining facility including virtual mine terrain and virtual mining trucks. Similar to ref. ^45^, we enable performance enhancement using data collected during real mining operations without the need for a lengthy pre-training phase. Real-world data are collected to supervise the sensor’s physical models in the virtual mine, refining the data to closely resemble real-world data.

We initialize the sensor models using their underlying principles first. For instance, the LiDAR sensor scans its surroundings by measuring the time of flight of laser pulses, which are reflected back if a target is encountered. Parameters of the emitted laser beams, including the azimuth and vertical angles, angle noise, and distance noise, can be directly modeled based on the sensor specifications. By simulating the intersection of the laser beam and the 3D environment model, laser point-cloud data can be generated. In addition, we project the three-dimensional grid into a depth map and determine the intersection of the laser beam and the depth map to simplify the computation.

When creating digital twins, a virtual intelligent vehicle would mirror the actions of its real counterpart in the real world, by which a set of virtual point-cloud data and real point-cloud data can be produced. By using real-world data as a form of supervision, we optimize the above parameters to better fit the real sensor models.

For simulating data under extreme weather, we manually introduce weather-related influence coefficients to transform the data domain. For LiDAR, we enhance the model by incorporating attenuation and backscattering coefficients to simulate the reception of pulse signals on foggy days. In addition, we leverage publicly available autonomous driving datasets collected on public roads to facilitate domain transfer of sensor data using CycleGAN^46^. This approach enables us to generate data corresponding to different weather conditions (e.g., rainy, foggy, and snowy) from mining data on sunny days. The loss function of the domain transfer

1 $$	J\left({E}_{1},{E}_{2},{G}_{1},{G}_{2},{D}_{1},{D}_{2}\right) \, = \, {\lambda }_{{{{{{{{\rm{v}}}}}}}}}\left({J}_{{{{{{{{\rm{vae}}}}}}}}}\left({E}_{1},{G}_{1}\right)+{J}_{{{{{{{{\rm{vae}}}}}}}}}\left({E}_{2},{G}_{2}\right)\right)\\ 	+{\lambda }_{{{{{{{{\rm{a}}}}}}}}}\left({J}_{{{{{{{{\rm{adv}}}}}}}}}\left({E}_{2},{G}_{1},{D}_{1}\right)+{J}_{{{{{{{{\rm{adv}}}}}}}}}\left({E}_{1},{G}_{2},{D}_{2}\right)\right)\\ 	+{\lambda }_{{{{{{{{\rm{c}}}}}}}}}\big({J}_{{{{{{{{\rm{cyc}}}}}}}}}\left({E}_{1},{G}_{1},{E}_{2},{G}_{2}\right) \\ 	+{J}_{{{{{{{{\rm{cyc}}}}}}}}}\left({E}_{2},{G}_{2},{E}_{1},{G}_{1}\right)\big)$$

where *E*_1_, *E*_2_, *G*_1_, *G*_2_, and *D*_1_, *D*_2_ refer to encoder, decoder, and discriminator, respectively. Subscript 1 corresponds to modules for virtual mining data, while subscript 2 corresponds to modules for real open road data. *J*_vae_, *J*_adv_, and *J*_cyc_ represent variational autoencoder loss, adversarial loss, and cycle-consistency loss, respectively, with *λ*_v_, *λ*_a_, *λ*_c_ as weights. When extreme weather actually occurs in the real mine, sets of virtual mining data on sunny days and real mining data under extreme weather are produced from digital twins, which are then utilized for supervised learning to optimize *E*_1_ and *G*_1_. The objective function is given below:

2 $$\theta^{\ast} = {\arg\min}_{\theta}\sum\limits_{t = 1}^{K}\left[ y(t,\theta) - {\hat{y}}(t)\right]^{T} \cdot W \cdot \left[ y(t,\theta) - {\hat{y}}(t)\right]$$

where $\hat{y}(t)$ and *y*(*t*, *θ*) represent the set of actual data and transferred virtual data, respectively, with *θ* denoting the network parameters of *E*_1_ and *G*_1_. The positive symmetric matrix *W* weights the importance of each output.

#### Predictive learning

Predictive learning involves constructing mapping relationships between mining operations and entities’ state transformation, which includes the motion trajectories of intelligent vehicles resulting from their movements, ground tracks, collisions, material handling, and data transmission. Real-world data is collected through sensors, and deep neural networks are employed to analyze and model this process.

In particular, we use sensors on intelligent vehicles and sensors installed in critical areas of the working sites, such as digging faces and berms, to gather information about the actions of intelligent vehicles and their impact on the mine. Differential GPS is used to collect vehicle trajectory data, while onboard cameras observe tracks and millimeter-wave radar monitors collisions. Cameras, radar, and onboard weight sensors are also used to monitor the progress of material loading and unloading. Moreover, we collect information on the reception of shared information during vehicle cooperation to simulate noise and delay in data transmission.

Regarding the modeling of actual vehicle motion trajectories, we model the regression relationship using a deep neural network with three hidden layers, each consisting of a Batch Normalization layer and a Rectified Linear Unit layer. The input is action sequences sampled from a short time window, while the output is sampled trajectory points. We employ differential GPS on actual vehicles to collect their trajectories, and we adjust them to a unified coordinate system at the starting position of the time window. We use the trajectory in the local coordinate system as the ground truth and train a regression model to map control actions to the actual resulting motion. Notably, we optimized the modeling of the dynamic kinematics using a data-driven approach to enhance accuracy and stability, which was initially constructed by simulation engineers using a model-driven approach.

#### Prescriptive learning

In the context of parallel intelligence, computational experiments generate massive amounts of data in a simulation system that employs both descriptive and predictive intelligence. Prescriptive intelligence, which refers to algorithms for specific mining operations, is generated as a result.

In order to accomplish this, we initially decompose the overall mining task into multi-level subtasks based on our experience in the mining field. We then develop a series of initialization algorithms, including obstacle detection, roadside detection, berm detection, route planning, arm trajectory planning, cooperative planning, balanced rolling, dynamic speed adjustment for uneven road surfaces, localization, and mapping, adaptive control, and multi-objective optimization-based scheduling, to create the first version of an autonomous transportation system. However, these initialization algorithms rely heavily on expert experience or are trained using a limited amount of field data, resulting in a lack of adaptability and instability over extended periods.

To address this issue, we first employ the initialization system to guide virtual vehicles in testing scenarios for computational experiments and then iterate the system operations using data-driven and reinforcement learning. For optimizing scheduling, we utilize a curriculum learning-based model to provide dense artificial rewards, with optimization objectives as evaluation metrics. We incorporated artificial rewards into the simulation reward sequence to create the basic structure of mixed training instances (“curriculum”), resulting in a more efficient scheduling method compared to previous multi-objective optimization. Second, we examined the adaptive task difficulty momentum enhancement strategy and used task difficulty feedback to adjust the experience weight adaptively, indirectly controlling the mixed “curriculum” difficulty. Finally, we sampled training data from the experience pool to construct different levels of “curriculum” data, updated the policy model, and achieved a more efficient and adaptive learning process.

### Scenario engineering for intelligent mining

Virtual mining scenarios are generated based on Scenarios Engineering Methodology^47^, providing a sufficient scenarios library for algorithm validation and optimization.

#### Scenario data form

We divide the mining transportation scenario into three layers, namely the mine environment, mining robots, and external conditions. The mine environment layer is comprised of 3D models and crucial semantic information resembling high-definition maps used in autonomous driving^48^. This information includes various details such as main roads, road edges, hanging walls, and area labels like loading sites, unloading sites, and intersections. mining robots, which refer to intelligent vehicles involved in the current scenario, make up the second layer. External conditions encompass environmental factors such as weather, lighting, and humidity, which can have a substantial impact on robot perception and control. For instance, poor visibility due to fog or low-light conditions can make it challenging for robots to perceive their surroundings and make accurate decisions. In addition, the scenario data also covers the interactions of robots, including both collaborative and competitive types. A mining scenario is a comprehensive reflection of the mining environment and intelligent vehicle behaviors in a particular spatial and temporal range.

To enable efficient storage and indexing of scenario data, we concatenate scenario element labels and action labels to generate scenario indexes. In particular, we employ graph convolutional networks to extract geographic information encoding from the semantic vector map, while weather and lighting conditions are classified from image data using convolutional neural networks. Furthermore, we generate action labels through a trained encoder network supervised by a human operator’s intention. The data are then stored in the scenario library based on the scenario index and difficulty rating obtained from scenario evaluation.

#### Scenario generation

A mining scenario comprises three types of elements and their interactions. To facilitate mining scenario generation, we maintain a repository of materials that includes a pool of mine models, a set of mining intelligent vehicle models, and a list of mining actions and external conditions.

Mine models are developed using data obtained from unmanned aerial vehicle scans, ground sensors installed at critical locations (such as landslide-prone areas, heading faces, and berms), and sensors on intelligent vehicles. While some methods do not necessitate complete 3D modeling of the environment and instead utilize network models to synthesize images from the desired viewpoint directly, we opted for a 3D model to achieve more interactive and user-friendly 3D visualization. Unmanned aerial vehicles fly around the mining area, gathering data through high-resolution color cameras, and structure-from-motion is employed to construct a complete model of the mine. During this process, several control points are established on the ground to align the coordinate system, scale, and adjust the model. Real-time data from ground sensors and sensors on intelligent vehicles are collected to monitor environmental changes and update the environment model accordingly. We have also adopted a semi-automated labeling approach to annotate the vector semantic layer of the mine model.

To create the intelligent vehicle model pool, we obtain kinematic models of different types of mining vehicles (e.g., mining trucks, excavators, and bulldozers), along with physical models of their sensors, using descriptive intelligence and predictive intelligence as a guide. We also incorporate time and weather conditions. A set of action tags is defined, including digging, dumping, loading, overtaking, changing lanes, accelerating, decelerating, backward, turning, stopping, and starting.

With the scenario elements and actions defined, we can deploy “training vehicles” into the virtual mining engine to create an adversarial environment and thus optimize-related algorithms, inspired by Feng et al.^49^. The virtual mining engine, which encompasses descriptive intelligence, predictive intelligence, and prescriptive intelligence, can simulate perception data and the impact of actions in specific scenarios, as well as direct the actions of “ladder players” to accomplish the intended objectives.

#### Scenario evaluation

In order to evaluate task performance across a range of scenarios, it is important to develop evaluation indicators that take into account factors such as safety, efficiency, and energy consumption. To this end, we have designed a set of task metrics^2^ for subtasks, including perception, localization, and scheduling, drawing on the expertise of specialists in the field.

Similar to ref. ^50^, we conducted an assessment of task complexities for seeking challenging testing tasks. A key insight is that the effectiveness of an algorithm in task evaluation can provide an indication of the simplicity of the corresponding scenario. As such, performance evaluation can also be used as a metric for assessing scenario difficulty. To facilitate this evaluation, we have divided scenarios into five levels of difficulty, ranging from easy to hard, which is shown below:

3 $${{{{{{{\rm{rank}}}}}}}}={{{{{{{\rm{INT}}}}}}}}\left({{{{{{{{\rm{F}}}}}}}}}_{{{{{{{{\rm{X}}}}}}}}}(s)* 5\right)$$

where *s* represents a score of task performance, F_X_() represents the cumulative probability density function of performance scores, and INT() represents the rounding function. To simplify the problem, we assume that the performance scores follow a Gaussian distribution.

We develop a classification model for scenario evaluation by utilizing scenarios and their corresponding difficulty levels obtained from actual mining operations. The model takes scenario encoding as input and outputs the corresponding scenario difficulty levels. By applying this model, virtual scenarios can be automatically classified, leading to the construction of a comprehensive scenario library.

### Supplementary information

Supplementary Discussion

Description of Additional Supplementary Files

Supplementary Movie 1

Supplementary Movie 2

Supplementary Movie 3

## Data Availability

Data can be accessed through our team’s publicly released dataset, AutoMine^2^, with additional data available from the authors upon reasonable request.

## Code availability

Code available from the authors upon reasonable request.
