# Supplementary material for: Autonomous mining through cooperative driving and operations enabled by parallel intelligence
Source: Commun Eng. 2024 May 31;3:75. doi: 10.1038/s44172-024-00220-5 (PMC11143282; doi:10.1038/s44172-024-00220-5)
Supplement: Supplementary file 1 — Supplementary Discussion [file 44172_2024_220_MOESM1_ESM.pdf]

Supplementary Materials for

Autonomous Mining Through Cooperative Driving and Operations Enabled by Parallel Intelligence

This PDF file includes:

Supplementary Discussion: Application of YuGong in Real Mines
Supplementary Table 1. Details on 9 representative mines deployed with YuGong
Supplementary Figure 1. Application of YuGong in 9 representative mines
Supplementary Figure 2. The hardware configuration of mining devices in YuGong

Supplementary Discussion

Application of YuGong in Real Mines

This material provides additional information on the successful implementation of the YuGong system in **over 30 mines** with various complex environments. The system has completed more than **4.1 million kilometers of mileage** without causing any major accidents in these mines. The detailed information on several representative mines is presented in Supplementary Table 1, while an overview of them can be seen in Supplementary Figure 1. Additionally, the sensor layout of the mining devices is illustrated in Supplementary Figure 2 to demonstrate how the sensor system collects environmental data. The system has demonstrated several advantages in these practices.

- It has been successfully applied in **open-pit mines** as well as **shaft mines** where GPS is denied.
- It can adapt to extreme climatic conditions such as cold areas with temperatures as low as minus 40 degrees Celsius, and humid and rainy areas, ensuring stable operation and safety.
- It supports both **fully unmanned and mixed manned-unmanned operations** that can be flexibly switched according to different scenarios and needs.
- It has been equipped on multiple types of heavy trucks ranging from 60 to 363 tons of various models and brands, achieving interconnection and collaboration among devices.
- In a single mine, it has achieved **a maximum scale of over 56 intelligent vehicles** that can be remotely managed and their data analyzed through a cloud platform.

In summary, the YuGong system is highly versatile, reliable, flexible, scalable and has shown significant advantages and potential in autonomous mining.

**Mine**	**Type**	**Environmental Characteristics**	**Scale of Autonomous Mining**
Baorixile	Open-pit Coal Ore	Extreme cold (-40°C and below); large mining area covering 50.72 km²	24/7 three-shift unmanned mining with 32 intelligent vehicles
Pingshuo East	Open-pit Coal Ore	Large mining area covering 48.73 km²	5 manned-unmanned mixed shifts; each consists of 1 electric shovel, 7 unmanned trucks, and N manned auxiliary equipment
Zhunneng	Open-pit Coal Ore	The largest Open-pit mine in Asia covering 28.35 hectares	3 unmanned shifts; largest formation consists of 36 intelligent vehicles
X.B.D.	Shaft Coal Ore	Subterranean; weak lighting and water vapor interference	Autonomous mining in underground tunnels
Hualian Zinc-Indium	Open-pit Zinc-Indium Ore	Rainy and foggy weather (low visibility)	Continuous operation of 5 autonomous trucks without safety inspectors
C.M.S Copper Mine	Open-pit Copper Ore	Humid climate; muddy roads	Full process operations without safety inspectors.
Huolinhe North	Open-pit Coal Ore	High altitude and alpine climate; long periods of snow cover	Dispatch management of unmanned/manned/mixed operations, involving 200 equipment
Huasheng Cement	Open-pit Cement Ore	Dusty environment	Full autonomous process of "mining-transportation-handling"
H.S.Q.	Open-pit Iron Ore	Strong sunlight in Summer; overexposure	8 unmanned vehicles and 20 manned vehicles; 24/7 mixed operations

Supplementary Table 1: Details on 9 representative mines deployed with YuGong

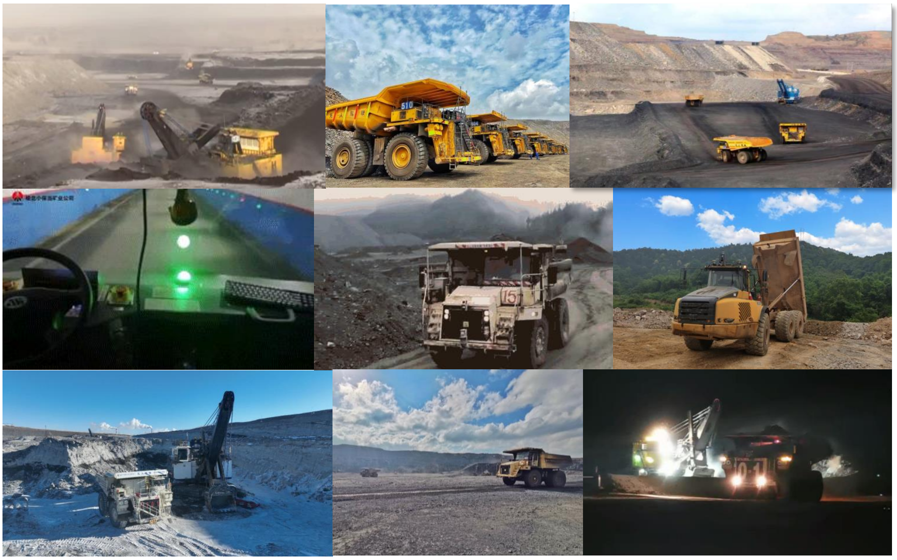

Supplementary Figure 1: Application of YuGong in 9 representative mines

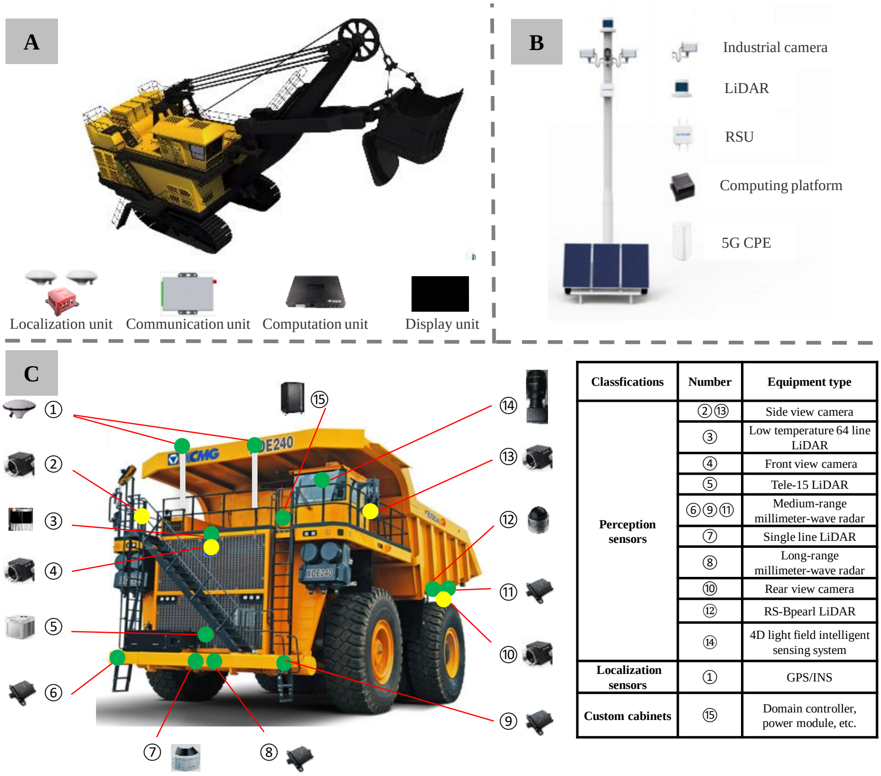

Classfications	Number	Equipment type
Perception sensors	②⑬	Side view camera
	③	Low temperature 64 line LiDAR
	④	Front view camera
	⑤	Tele-15 LiDAR
	⑥⑨⑪	Medium-range millimeter-wave radar
	⑦	Single line LiDAR
	⑧	Long-range millimeter-wave radar
	⑩	Rear view camera
	⑫	RS-Bpearl LiDAR
	⑭	4D light field intelligent sensing system
Localization sensors	①	GPS/INS
Custom cabinets	⑮	Domain controller, power module, etc.

Supplementary Figure 2: **The hardware configuration of mining devices in YuGong** (LiDAR: Light Detection and Ranging, RSU: Road Side Unit, 5G CPE: 5G Customer Premises Equipment, INS: Inertial Navigation System). **A)** The semi-autonomous excavators **B)** The intelligent roadside infrastructure **C)** The autonomous truck.
