# Supplementary material for: Autonomous mining through cooperative driving and operations enabled by parallel intelligence
Source: Commun Eng. 2024 May 31;3:75. doi: 10.1038/s44172-024-00220-5 (PMC11143282; doi:10.1038/s44172-024-00220-5)
Supplement: Supplementary file 2 — Description of Additional Supplementary Files [file 44172_2024_220_MOESM2_ESM.pdf]

Description of Additional Supplementary Files

File name: Supplementary Movie 1

Description: Real-World Demonstration of Autonomous Mining in Open-Pits

File name: Supplementary Movie 2

Description: A Showcase of the Simulation

File name: Supplementary Movie 3

Description: Operational Performance in Adverse Conditions
